# Correlation between Thrombus Perviousness and Distal Embolization during Mechanical Thrombectomy in Acute Stroke

**DOI:** 10.3390/diagnostics13030431

**Published:** 2023-01-25

**Authors:** Fabio Pilato, Iacopo Valente, Andrea M. Alexandre, Rosalinda Calandrelli, Luca Scarcia, Francesco D’Argento, Emilio Lozupone, Vincenzo Arena, Alessandro Pedicelli

**Affiliations:** 1 Department of Medicine and Surgery, Unit of Neurology, Neurophysiology, Neurobiology and Psichiatry, Università Campus Bio-Medico di Roma, Via Alvaro del Portillo, 00128 Roma, Italy; 2 Operative Research Unit of Neurology, Fondazione Policlinico Universitario Campus Bio-Medico, Via Alvaro del Portillo, 00128 Roma, Italy; 3 UOC Radiologia e Neuroradiologia, Polo Diagnostica Per Immagini, Radioterapia, Oncologia ed Ematologia, Fondazione Policlinico Universitario Agostino Gemelli IRCCS, Roma—Area Diagnostica Per Immagini, 00168 Rome, Italy; 4 Department of Neuroradiology, Vito Fazzi Hospital, 73100 Lecce, Italy; 5 Istituto di Anatomia Patologica, Dipartimento Scienze della Salute della Donna, del Bambino e di Sanità Pubblica-Area Anatomia Patologica-Fondazione Policlinico Universitario Agostino Gemelli IRCCS, 00168 Roma, Italy

**Keywords:** acute ischemic stroke, thrombus perviousness, clot histology, computed tomography, multiphase computed tomography angiography, thrombectomy, rt-PA, distal embolization

## Abstract

Purpose: Thrombus permeability has been related to clot composition and treatment outcomes in stroke patients undergoing reperfusion therapies. The aim of this study was to evaluate whether thrombus perviousness, evaluated by multiphase computed tomography angiography (mCTA), is associated with distal embolization risk. Methods: We interrogated our dataset of acute ischemic stroke (AIS) patients involving the M1 segment of the middle cerebral artery (MCA) who had undergone mechanical thrombectomy, and we calculated thrombus average attenuation measurement (dHU) on non-contrast CT (NCCT) and clot perviousness on mCTA. dHU was calculated as the difference between the thrombus HU average value (tHU) and the HU average value on the contralateral side (cHU), while perviousness was calculated as the difference in mean clot density on mCTA and NCCT both in arterial (Perviousness pre-post-1) and delayed (Perviousness pre-post 2) phases. Results: A total of 100 patients (53 females (53%), mean age 72.74 [± 2.31]) with M1 occlusion were available for analysis. Perviousness, calculated between baseline and arterial phase of mCTA (Perviousness pre-post1), was lower in patients with distal embolization (*p* = 0.05), revealing an association between reduced perviousness and distal embolization risk. Logistic regression showed that thrombus perviousness calculated on the arterial phase of mCTA (OR, 0.66; 95% CI, 0.44–0.99] (*p* = 0.04)) and the contact aspiration technique (OR, 0.39; 95% CI, 0.15–1.02] (*p* = 0.05)) were protecting factors against distal embolization. Conclusion: Our study showed an association between reduced perviousness and distal embolization, suggesting that perviousness evaluation may be a useful neuroimaging biomarker in predicting distal embolization risk during mechanical thrombectomy.

## 1. Introduction

The occlusion of an artery in the brain, leading to hypoperfusion of the involved tissues, causes a stroke. Acute stroke management is centered on re-vascularizing arteries swiftly, and an early complete recanalization of the occluded artery is a strong predictor of favorable functional outcomes in patients with acute ischemic stroke [1]. Intravenous thrombolysis and endovascular thrombectomy have deeply redesigned the management of acute ischemic stroke, although stroke remains a leading cause of death and long-term disability [2,3]. The thrombus features represent a critical factor for successful reperfusion, although fast and complete reperfusion is also related to other factors such as vessel anatomy and technical struggles [4]. The study of thrombus composition is an interesting research field because it may provide some insights into thrombotic mechanisms and may also have potential therapeutic relevance [5]. However, thrombus composition can be studied only after the completion of thrombectomy; thus, thrombus imaging biomarkers obtained before thrombectomy constitute important factors capable of predicting thrombectomy recanalization efficacy [6,7]. Neuroimaging may provide some indirect insights about thrombus features. Several studies evaluated acute brain computed tomography (CT) imaging to assess thrombus characteristics, and these features were demonstrated to be useful imaging biomarkers for clot characterization, stroke pathogenesis, and outcome prediction [6,8,9,10,11,12]. Although several factors may influence distal embolization risk, thrombus histopathological and radiological features along with patients’ clinical features may concur to clot formation, fragmentation process, and embolization risk [13,14]. 

Thrombus permeability reflects the ability of soluble molecules to move within the gaps among adjacent platelets, fibrin filaments, and red blood cells, and clinical and preclinical studies have shown that thrombus permeability may influence the resistance of the thrombus [5,15].

Previous histopathological studies, in AIS patients with AIS, demonstrated that retrieved clots are highly heterogeneous [13], and recent data suggest that thrombus composition may impact mechanical thrombectomy outcome [6,13,14].

Neuroimaging using head non-contrast computed tomography (NCCT) and CT angiography (CTA), besides localizing the occlusion site, allows the direct assessment of the thrombus features such as length, density, and perviousness; these thrombus characteristics have been associated with recanalization rates and functional outcome [5,16,17]. Thrombus perviousness, derived from head CT performed at the early stage of stroke, is a promising biomarker of thrombus composition and resistance [5,10]. Previous studies about clot characteristics supported the usefulness of an automatic assessment of thrombus characteristics, and the CTA index was demonstrated to be a useful and easily available surrogate marker for assessing thrombus perviousness [18]. However, the relation between perviousness and distal embolization risk is still an understudied topic that may have relevant therapeutic significance. While some studies demonstrated the usefulness of thrombus perviousness analysis in predicting functional outcomes of intravenous recombinant tissue-type plasminogen activator (rt-PA) treatment [19], only a few studies evaluated its utility in predicting endovascular procedures efficacy [20] and thrombus propensity to distal embolization.

The aim of the present study was to evaluate whether thrombus perviousness evaluated on the CTA is associated with distal embolization risk. 

## 2. Methods

### 2.1. Patient Imaging

We performed a retrospective analysis of a prospectively maintained dataset of a comprehensive tertiary stroke center inclusive of all cases of endovascular treatment for acute ischemic stroke.

We included patients with the following characteristics: (1) age ≥18 years, (2) large vessel occlusion (LVO) involving the internal carotid artery (ICA) terminus and M1 segment of the middle cerebral artery (MCA), (3) acceptable image quality on NCCT and a multiphase CTA (mCTA) with arterial and venous phases, and (4) patients who underwent thrombectomy resulting in almost complete reperfusion (TICI 2b/3).

All patients underwent NCCT and mCTA at baseline. All eligible patients received IV rt-PA according to standard guidelines [21]; patients underwent mechanical thrombectomy when mCTA showed an LVO. Endovascular procedures were performed under general anesthesia or conscious sedation at the discretion of individual interventionalists. Mechanical thrombectomy was performed with a stent-retriever and proximal guide catheter aspiration, direct contact aspiration, or a combination of stent-retriever and distal aspiration. Baseline demographic, clinical, radiological, procedural, and histological variables were recorded. Regarding patients’ baseline data, we considered age, gender, smoking, diabetes, hypertension history, and AF. Distal embolization was defined as the presence of distal branch occlusion of the same vascular territory or new vascular territories during thrombectomy.

This study was approved by the local Institutional Review Board.

### 2.2. Image Analysis 

Baseline CT imaging was performed using a 64-multislice CT (GE MEDICAL SYSTEM Optima CT660 645). Acquisition parameters for NCCT were 120 kv and 44 mAs, and acquisition duration was 9 s. Acquisition parameters for mCTA were 100 kV and 4 mAs, and acquisition duration was 21 s; after administration of 80 mL of contrast media at 4 mL/s flow, the acquisition was composed of three subsequent phases (separated by an interval of 8 s): the first phase (acquired in the arterial phase) extends from the aortic arch to the vertex and the next two phases (acquired in the early and late venous phases) from occipital foramen to vertex. 

The following radiological thrombus characteristics on baseline CT scans were assessed: thrombus length, thrombus density, and thrombus perviousness.

#### Thrombus Attenuation and Length 

**Figure 1:**
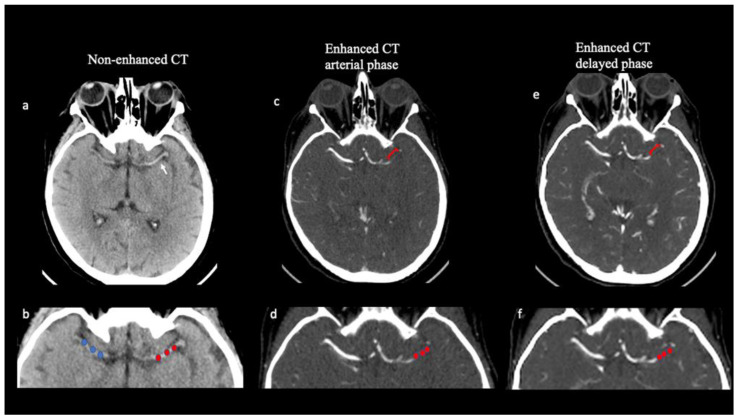
Thrombus attenuation measurements and thrombus perviousness quantification. Axial non-contrast CT (NCCT; **a**,**b**) demonstrates a hyperattenuating (“dense”) middle cerebral artery (MCA) (white arrow in **a**) in a 67-year-old patient with acute stroke. Thrombus attenuation measurement was calculated on NCCT as the difference between thrombus average density and contralateral artery by placing 3 spherical ROIs in the proximal, middle, and distal parts of the thrombus in the M1 segment of the left middle cerebral artery (red ROIs in **b**) and along the same vessel on the contralateral side (blue ROIs in **b**). Multiphase CTA (mCTA; **c**–**f**) identifies occlusion length of the M1 segment of the left middle cerebral artery, demonstrated as contrast-filling defects (square bracket in **c**,**e**). Three regions of interest (red ROIs) were placed in a left middle cerebral artery thrombus both in the arterial (**c**,**d**) and in the delayed phases (**e**,**f**) of the mCTA. Clot perviousness measurement was calculated as the difference in average thrombus attenuation measured on the arterial or delayed phase of mCTA and on NCCT.

On NCCT imaging, the thrombus HU value was calculated by placing three spherical regions of interest (ROIs) within the thrombus location (size 3 × 3 voxels) (proximal, middle, and distal clot segment) but away from the boundaries of the thrombus (Figure 1). HU values were also calculated, using ROIs placed in three corresponding locations, on the contralateral artery to correct for the variability of hematocrit levels. The mean thrombus attenuation on NCCT was calculated by averaging the HU of the three ROIs both in the thrombosed and the contralateral arteries. On NCCT, thrombus average attenuation measurement (dHU) was calculated as the difference between the thrombus HU average value (tHU) and the HU average value on the contralateral side (cHU). 

The following equation was applied: [dHU = tHU − cHU]. 

The occlusion extension, defined as clot length, was determined based on the contrast-filling defects between the proximal and distal thrombus borders found on the delayed phase of CTA. Delayed phase images were chosen because they allowed the maximum amount of time to allow a contrast to reach the distal face of the clot from leptomeningeal collaterals. Mural calcifications were excluded from the analysis (Figure 1). 

### 2.3. Estimation of Perviousness

Thrombus HU average value was calculated both on NCCT and mCTA imaging. The clot perviousness measures the contrast penetration into a thrombus [20]. To estimate contrast penetration into the thrombus, the increase in the mean HU of the thrombus on mCTA was compared with the mean HU of the clot on NCCT. In the mCTA, two measurements of the average thrombus density were performed in the arterial and delayed phases, respectively, using the same method. Thrombus attenuation was measured both for the arterial (Perviousness pre-post 1) and delayed (Perviousness pre-post 2) phase of the mCTA (Figure 1). 

Partial occlusions (thrombi occluding 50% of the vessel diameter), bilateral thrombi, too short thrombi (<2 mm), thrombi very close to the bone (affected by partial volume/blooming artifact of bone), and/or calcified emboli were also excluded.

Two experienced neuroradiologists, blinded to clinical information and clot histology, reviewed the images.

### 2.4. Histological Analysis of Clots 

Histological analysis of the clots was performed according to the previously described analysis [6].

### 2.5. Statistical Analysis

For continuous measures, means and standard deviation (SD) or median with interquartile range (IQR) are shown; for categorical measures, frequencies, and percentages are presented.

The primary outcome was the presence of distal embolization during mechanical thrombectomy. Differences between the two groups were tested with the χ2 or two-tailed Fisher’s exact test for binary variables, and with the t-test or Mann–Whitney U test for continuous variables, as appropriate. In order to evaluate the determinants of distal embolization, we applied a logistic regression model including pre-specified clinically relevant variables (thrombus length at CTA, rt-PA administration, and technique used for mechanical thrombectomy) and all significant (*p* ≤ 0.05) variables included in the univariate analysis. Odds ratios (OR) and 95% confidence intervals (95%CI) were reported. 

Statistical analysis was performed with STATA 15.1 (StataCorp LLC – 4905 Lakeway Dr, College Station, TX, USA).

## 3. Results

Baseline demographics, clinical, radiological, and procedural data are reported in Table 1.

A total of 100 patients (53 females (53%), mean age 72.74 (SD ± 2.31)) with M1 occlusion were available for analysis. A total of 99 patients (99%) had a stroke involving only anterior circulation, 28 patients (28%) had diabetes, and 53 patients (53%) had a cardioembolic stroke. There were 45 patients (45%) with distal embolization to distal branches of the same territory of primary occlusion or embolism in new territories during thrombectomy. No difference between groups was found for demographic features and treatments (*p* > 0.05). Fifty-two patients (52.0%) underwent combined endovascular techniques, whereas forty-five (45.0%) underwent contact aspiration and only three (3.0%) underwent stent-retriever thrombectomy alone (*p* > 0.05). Forty-nine patients (49.0%) underwent rt-PA administration before endovascular thrombectomy. No difference was found between the groups in histopathologic clot composition (*p* > 0.05). 

The admission NCCT scan showed hyperdense-thrombus signs in sixty-one patients (61.0%), and the median thrombus length at CTA was 14.57 mm (IQR 12.83–16.31). In the group showing distal embolization, a trend toward a longer length of the thrombus emerged (16.05 mm) (IQR 13.01–19.08) compared with the group without distal embolization (13.22 mm) (IQR 11.44–15.00) (*p* = 0.11).

Perviousness, calculated between baseline and arterial phase of mCTA (“Perviousness Pre-Post 1”) was lower in patients with distal embolization (*p* = 0.05), revealing an association between arterial-phase perviousness and distal embolization. 

### Predictors of Distal Embolization

Logistic regression including rt-PA, thrombus length at CTA, mechanical thrombectomy technique, and perviousness pre-post 1 as independent variables showed that thrombus perviousness calculated on the arterial phase of mCTA (“Pre-Post 1”) (OR, 0.66; 95% CI, 0.44–0.99] (*p* = 0.04)) and contact aspiration technique (OR, 0.39; 95% CI, 0.15–1.02; ref. Combined technique] (*p* = 0.05)) were protecting factors against distal embolization (Table 2).

## 4. Discussion

In acute stroke patients, neuroimaging is mandatory before thrombectomy, and it may also provide supportive information about arterial anatomy and thrombus location [21,22,23]. Neuroimaging evaluation may also have prognostic relevance in terms of recanalization and outcome [19,20]; some studies used baseline CT as a surrogate biomarker of thrombus composition [10].

Several studies carried out on clots evaluated perviousness, composition, and clinical outcome, but the relationship between thrombus composition and perviousness evaluated by mCTA-NCCT subtraction remains debated. Permeable clots may not fully occlude the artery, allowing perfusion of the brain tissue distal to the clot, and they may be more prone to dissolution after thrombolysis [19] and easily removed by thrombectomy; moreover, permeability may be related to clot structural properties [20], and these properties may be assessed indirectly by means of thrombus perviousness evaluation [5].

Recently, there has been considerable interest in studying thrombus composition and its effect on both thrombolysis and thrombectomy [5,19]. 

Some studies evaluated perviousness and the first-pass effect by stent retriever [20], but literature findings are conflicting because a number of factors may play a role in this process [14].

This study sought to evaluate whether thrombus perviousness, evaluated on baseline CT and mCTA, might be a suitable tool to predict the risk of distal embolization. We studied patients with LVO stroke involving anterior circulation and we evaluated pre-selected factors linked to distal embolization risk.

Our data showed that thrombus perviousness, evaluated on the arterial phase of mCTA, was related to a reduced risk of distal embolization, but the differences in composition among removed clots were not significant. Moreover, thrombolysis and onset-to-needle time were not associated with distal embolization risk. 

According to previous studies, perviousness determined on the arterial phase of mCTA is more suitable than perviousness determined on the delayed phase of mCTA in the evaluation of outcomes because it better reflects the thrombus permeability in patients with acute ischemic stroke [18]. Some studies regarding clot composition and thrombus imaging demonstrated that imaging may vary depending on different types of clot composition [10,24,25]. Moreover, some studies examined the relationship between the degree of perviousness and thrombus histology, but no univocal results emerged. In a study [9], clot permeability was significantly linked to a lower fraction of red blood cells and more fibrin and platelet content. On the other hand, in another study [10], thrombus perviousness was associated with higher red blood cell density and lower fibrin density, and these differences had already been reported [26].

In accordance with previous studies, we found that removed clots were heterogeneous in their composition [14], in terms of stratification and crystal composition, but no relationship between clot permeability and clot composition was found [26]. These data suggest that not only do clot features impact clot permeability, but a number of other factors probably related to the artery and patient may be responsible for the contrast penetrating a thrombus (e.g., vessel permeability, local pressure, composition, retraction, clot length, and location) [14], and all these factors may be evaluated by thrombus imaging.

Previous studies demonstrated that thrombus length was associated with distal embolization risk [6], whereas other studies found that clot volume, more than clot length [17], could be related to the effectiveness of mechanical thrombectomy and systemic thrombolysis [26]. Our analysis shows a trend towards distal embolization in patients with a longer length of thrombus; the difference was not significant but because of the small sample size an effect may not be ruled out. 

Some studies evaluated the predictors of the first-pass effect; the perviousness was not found related to the first-pass effect during thrombectomy using a stent retriever [4,20]. We assessed the relationship between perviousness and distal embolization, but we did not evaluate the first-pass effect or the number of attempts to reach recanalization. 

Our data showed that thrombus perviousness was related to a reduced risk of distal embolization. Moreover, we found that as far as technique is concerned, contact aspiration may have a reduced risk of distal embolization. This effect may be related to the peculiar technique used during aspiration because the clot is not passed through, as the stent retriever requires, but is only aspirated by contact. However, a combination of these effects is conceivable, although our data do not allow us to weigh this difference. 

A possible explanation of these findings is that harder thrombi, thus thrombi with lesser perviousness and thrombi with a longer length, are more difficult to remove [14], whereas the increased clot perviousness may be related to a higher propensity for removal because of clot softness. Removal propensity might not only be dependent on histological clot composition but also on local factors related to endothelium or arterial wall. Undoubtedly, a combination of clot-related, patient-related, and technique-related features may have a potential combined effect in reducing distal embolization, although these are still understudied factors, and further studies with larger sample sizes are needed to address this point. 

Studies about mechanical thrombectomy outcomes are challenging because they depend not only on data about thrombus and patients’ features, but also on other variables linked to operators, technical procedures, and institutional protocols. Multicenter studies allow reaching a higher number of enrolled patients, making results more trusted, but variables linked to operators and procedures may be prevalent, counterbalancing the benefits of a larger sample size. On the other hand, a single-center study may avoid this shortcoming, reducing the number of variables and demanding a smaller sample size, although results should be taken with caution before being confirmed by prospective studies.

Thrombus research has contributed to developing novel devices and strategies to identify and retrieve the thrombus [14]. These understandings may lead to a tailored approach to stroke patients based on baseline thrombus features, with the aim to increase the effectiveness of the treatment. Clot perviousness seems to be an important imaging biomarker and seems to have potential therapeutic relevance in AIS patients, although studies on its influence on mechanical thrombectomy outcomes are still ongoing and their results are not definitive yet. We are aware that the retrospective design and the small sample size may be a study limitation, and further prospective studies, with a multicenter design, are needed to confirm these results.

In conclusion, our data showed an association between reduced perviousness and distal embolization risk during mechanical thrombectomy, suggesting that perviousness evaluation may be a useful neuroimaging tool in predicting distal embolization risk.

## Figures and Tables

**Table 1:** Distal Embolization.

Variables	No Distal Embolization (*n* = 55)	Distal Embolization (*n* = 45)	Total (100)	*p*-Value
Age (years)	74.18 (71.30–77.07)	70.97 (67.29–74.66)	72.74 (70.43–75.04)	0.17
Female *n* (%)	25 (45.45%)	28 (62.22%)	53 (53.00%)	0.09
Diabetes *n* (%)	15 (27.27%)	13 (28.89%)	28 (28.00%)	0.86
AF *n* (%)	17 (30.91%)	12 (26.67%)	29 (29.00%)	0.64
Smoking *n* (%)	9 (16.36%)	8 (17.78%)	17 (17.00%)	0.85
Hypertension *n* (%)	43 (78.18%)	28 (62.22%)	741 (71.00%)	0.08
Systolic Pressure (mmHg)	141.62 (130.42–152.81)	136.07 (126.96–145.18)	138.69 (131.57–145.81)	0.44
Diastolic Pressure (mmHg)	81.38 (75.81–86.96)	78.14 (72.40–83.88)	79.67 (75.67–83.67)	0.42
Mean Pressure (mmHg)	105.02 (96.80–113.24)	98.12 (91.75–104.50)	100.79 (95.71–105.87)	0.19
Wake-up-Stroke *n* (%)	7 (12.73%)	3 (6.67%)	10 (10.00%)	0.31
rt-PA *n* (%)	26 (47.27%)	23 (51.11%)	49 (49.00%)	0.70
Onset-to-Needle (min)	154.17 (121.48–186.85)	168.14 (120.26–216.01)	160.85 (132.55–189.15)	0.62
Cardioembolic stroke *n* (%)	30 (60.00%)	23 (62.16%)	53 (53%)	0.84
Thrombus Length at CTA (mm)	13.22 (11.44–15.00)	16.05 (13.01–19.08)	14.57 (12.83–16.31)	0.11
Hyperdensity at CT *n* (%)	30 (54.55%)	31 (68.89%)	61 (61.00%)	0.14
Occlusion site ad DSA				0.45
ICA	13 (24.07%)	16 (34.78%)	29 (29.00%)	
M1	32 (59.26%)	24 (52.17%)	56 (56.00%)	
ICA+M1	5 (9.26%)	5 (10.87%)	10 (10.00%	
M2	4 (7.41%)	1 (2.17%)	5 (5.00%)	
HCT (%)	39.54 (38.06–41.02)	39.95 (38.55–41.34)	39.72 (38.70–40.75)	0.70
*n*. of Passages	1.96 (1.58–2.35)	2.09 (1.72–2.46)	2.02 (1.75–2.29)	0.65
Technique *n* (%)				0.20
Combined	25 (45.45%)	27 (60.00%)	52 (52.00%)	
Stent-retriever	1 (1.82%)	2 (4.44%)	3 (3.00%)	
Contact Aspiration	29 (52.73%)	16 (35.56%)	45 (45.00%)	
HU pre-CE	53.04 (50.84–55.24)	54.71 (52.34–57.09)	53.86 (52.24–55.48)	0.31
HU post-CE 1	57.25 (54.96–59.54)	58.70 (56.31–61.09)	57.96 (56.31–59.61)	0.39
HU post-CE 2	61.95 (59.54–64.36)	62.56 (60.03–65.09)	62.25 (60.51–63.98)	0.73
Perviousness pre-post 1	4.24 (3.89–4.58)	3.71 (3.33–4.10)	3.98 (3.72–4.25)	0.05
Perviousness pre-post 2	12.49 (10.01–14.98)	12.32 (9.76–14.87)	12.41 (10.64–14.18)	0.92
Fragment Dimension (mm)	12.00 (9.40–14.60)	11.72 (9.63–13.81)	11.37 (10.03–12.72)	0.87
Mastocytes *n* (%)	1 (16.67%)	5 (50.00%)	6 (37.50%)	0.18
Stratification *n* (%)	27 (64.29%)	22 (51.16%)	49 (57.65%)	0.22
Chrystals *n* (%)	2 (4.76%)	2 (4.65%)	4 (4.71%)	0.98
Heritrocitic Core *n* (%)	26 (61.90%)	22 (51.16%)	48 (56.47%)	0.32

*p*-values by *t*-test for continuous variables and χ2 test for binary/categorical variables. AF: atrial fibrillation; rt-PA: intravenous recombinant tissue-type plasminogen activator; HCT: hematocrit.

**Table 2:** Logistic regression.

Distal Embolization	Coef.	*p*-Value	95% Conf	Interval
rt-PA	1.07	0.89	0.41	2.8
Thrombus Length at CTA	1.04	0.21	0.98	1.11
Technique: (ref. Combined)	1			
Contact Aspiration	0.39	0.05	0.15	1.02
Perviousness pre-post 1	0.66	0.04	0.44	0.99

## Data Availability

The data that support the findings of this study are available from the corresponding author, [F.P.], upon reasonable request.
